# INTEREST GROUP SESSION—HEALTH BEHAVIOR CHANGE: USING DATA ABOUT PATIENT CHARACTERISTICS TO IMPROVE FALL PREVENTION

**DOI:** 10.1093/geroni/igz038.2091

**Published:** 2019-11-08

**Authors:** Gwen Bergen

**Affiliations:** Centers for Disease Control and Prevention, National Center for Injury Prevention and Control, Division of Unintentional Injury Prevention, Atlanta, Georgia, United States

## Abstract

Over one in four U.S. older adults (age 65+) reports falling each year with fall-related medical costs estimated at $50 billion. The American Geriatrics Society/British Geriatrics Society Clinical Practice Guideline for Prevention of Falls in Older Persons recommends that healthcare providers assess and manage their patients’ fall risk. The Centers for Disease Control and Prevention’s Stopping Elderly Accidents, Deaths, and Injuries (STEADI) initiative helps healthcare providers incorporate these guidelines by providing tools on how to screen, assess, and intervene to reduce risk. Evaluations of fall prevention have focused on the clinical process and outcomes. Understanding clinical activities is important in fall prevention but a better understanding of older adult characteristics that increase fall-risk, and attitudes that may affect their adoption of evidence-based interventions could improve the effectiveness of prevention strategies. The five presentations in this session include: 1. Demographic, health and functional characteristics of older adults with increased fall risk. 2. Caregivers of people with chronic conditions or disability as a group with increased fall risk. 3. The most effective and efficient ways of identifying older adults with increased fall risk. 4. Facilitators and barriers to older adults’ adherence to evidence-based fall interventions. 5. Applying knowledge of older adult attitudes to improving an implementation of STEADI-based fall prevention. Multifactorial fall prevention strategies such as STEADI focus on the clinical aspects of fall prevention but their success depends on understanding and incorporating older adult characteristics and attitudes. The information presented in this session can inform fall prevention strategies and improve health.

